# Functions of the Nervous System

**Published:** 1858-06

**Authors:** C. B. Chapman


### FUNCTIONS OF THE NERVOUS SYSTEM.

BY PROF. C. B. CHAPMAN.

### THE MEDULLA OBLONGATA.

In this series of articles, the animal system of nerve centers, the *cerebrum*, the *cerebellum*, and the *spinal cord*, as well at the nerves which pass out from the cord, and from the cranial cavity in pairs, have in turn been referred to. It remains to discribe an appendage of these several centers, which pertains to all of them.

Within the cranium, and resting upon the basilar process of the occipital bone, is that cranial prolongation of the spinal chord, which brings it not only into relation, but into direct functional connection with the encephater centers. This body may be distinguished by neuclei of gray matter, and by a peculiar arrangement of fibrous bands by which it is connected with these several centers, as well as by the distribution and endowment of the nerves which are connected with it. The boundary of that body is at the pons varolii above, while it terminates below at the foramen magnum of the occipital bone. This appendage can not be described in a satisfactory way without the use of models or drawings, in order that the eye may be addressed. Still a simple description may avail in acquiring a view of its connections, and functional relations with the large centers.

The *medulla oblongata* has columns projecting, before and behind which are continuations of the anterior and posterior columns of the spinal cord. These columns terminate above in the form of pyramids, and assume the names in front of *corpora pyramidalia*, and those behind, which are also partly superior and less prominent, are called *corpora pyramidalia posterior*.

The office of the medulla ablongata, seems to be that of connecting these three nerve centers, (viz : the cerebrum, the cerebellum and the spinal cord,) which it does by a continuation of its fibres into all of them, in such way as secures to them simultaneous and harmonious action.

A brief recapitulation of the offices of these centers may not be out of place.

The spinal cord alone performs the function of sensation, and endows the being with the power of motion.

These offices are performed through the agency of two pairs of columns, which are quite distinct in situation, although in near relation. The centers which occupy the cranial cavity, are also entirely distinct in their functions, one of them imparting the faculty of combining muscular motion, or of securing harmony in muscular action, while the other gives direction to all actions through the exercise of the intellectual faculties.

It will be seen that an animal which possesses the spinal center alone, is only endowed with sensation and motion, which last is only performed under the influence of a blind impulse. A being which, in addition to this, is endowed with a cerebellum, can only perform motions with more precision, but not with a purpose. When a cerebrum is superadded, the being can perform actions under the influence of motive, or in a way that will secure the accomplishment of a definite object. In other words, when the three centers are harmoniously combined, the cerebrum commands the spinal cord to perform certain and definite offices. These would be but poorly performed, but for the interposition of the cerebellum, which enables this center to ensure the obedience of the spinal cord to its mandates, by securing the combined and harmonious action of the muscles. Either of these centers may become impaired or rendered useless, while the others may perform their independent office, but the diminished value of such functions, thus performed, will be readily appreciated by the philosophic student, for of what avail, intellectually considered, would a perfect cerebrum be, without a spinal center. The being could still think, but such thought could secure no action. Even the faculty of speech, as well as other common means of communication, which are peculiar to intelligent beings, would not be available, so that such possession would only add to his degradation. The spinal center, too, may perform the only active nerve function in a human being, to one example of which I have already referred, whose life was maintained through a succession of years, by the agency of a cerebrum that belonged to another organism, or by the preparation and administration of food by another person.

The *ganglionic* or *sympathetic*, which is also sometimes called the *nutritive*, to distinguish it from the *animal system*, performs an office quite diverse from those before described. This system seems to preside over the function of nutrition in animals, and in the lowest animals is the only system which exists. It has for its centers in the higher class of animals, on the vestetrules a succession of knots, or gangliæ (which gives the system a name) that are situated in the larger cavities near the spinal column. In the inferior grades, these gangliæ are situated in relation with the nutritive apparatus; viz : the digestive and circulating organs, and where the lowest development exists, as in many articulates, it is merely annular form in relation with the Œsophagus.

